# Adhesion characteristics of solution treated environmental dust

**DOI:** 10.1038/s41598-020-70858-6

**Published:** 2020-08-14

**Authors:** Johnny Ebaika Adukwu, Bekir Sami Yilbas, Almaz S. Jalilov, H. Al-Qahtani, Mubarak Yaqubu, Abba Abdulhamid Abubakar, Mazen Khaled

**Affiliations:** 1 Mechanical Engineering Department, KFUPM, Dhahran, Saudi Arabia; 2 K.A.CARE Energy Research and Innovation Center, DTV, Dhahran, 31261 Saudi Arabia; 3 Center of Research Excellence in Renewable Energy (CoRE-RE), KFUPM, Dhahran, Saudi Arabia; 4 Chemistry Department, KFUPM, Dhahran, Saudi Arabia; 5 Chemistry and Earth Sciences, Department, Qatar University, Qatar, Saudi Arabia

**Keywords:** Mechanical engineering, Fluids

## Abstract

Environmental dust is modified towards self-cleaning applications under the gravitational influence. Dust particles are collected in the local area of Dammam in Saudi Arabia and they are treated with a dilute hydrofluoric acid solution. The changes in chemical and adhesion characteristics of the dust particles prior and after the solution treatment are analyzed. Force of adhesion and work required to remove dust from hydrophobic and hydrophilic glass surfaces are assessed, separately, for solution treated and collected dust. We show that aqueous hydrofluoric acid solution treatment modifies some dust components while causing the formation of submicron cracks and nano/submicron porous/pillars like textures on the dust particles. The texture generated on dust surfaces after the solution treatment has a great influence on dust adhesion characteristics. Hence, the solution treated dust particles result in lower adhesion on hydrophobic and hydrophilic glass surfaces as compared to that of untreated dust. The gravitational force enables to remove solution treated dust from inclined glass surfaces, which becomes more apparent for hydrophobic surfaces.

## Introduction

Climate change causes increased cycles of dust storms and dust settlement on surfaces, especially in urban areas, significantly influences the urban life in terms of agriculture^1^, health^2^, renewable energy harvesting^3^, etc. Mitigation of dust from surfaces becomes one of the recent challenges and, particularly, dust removal from energy harvesting device surfaces becomes necessary towards sustaining device performance. Using the gravitational influence only removing dust from surfaces becomes fruitful. The inclination of the surface can create the gravitational influence while causing dust particles displacement on the surface^4^. However, frictional and adhesion forces (such as van der Walls forces) between the particles and the surface generate a retardation effect on the motion of dust particles along the inclined surface. Since the dust particle size varies and large size particles can cause avalanche influence on the remaining small size dust particles while enhancing dust motion on the surface^4^. However, some compounds in the dust particles, such as alkaline and alkaline earth metal compounds, do not satisfy the stoichiometric elemental ratio^5^; hence, this causes ionic forces to be created on the particle surface while contributing to the interfacial force between the dust and inclined surfaces. This also enhances the pinning effect of the dust particles on the inclined surface. However, lowering surface energies of both the dust particles and the inclined surface, via chemical modification, can reduce the interfacial attraction between both surfaces. Therefore, investigation of dust particles removal from inclined surfaces with and without chemical modifications becomes essential for dust cleaning applications under the gravitational influence.

Dust composes of several elements and compounds, which depend on the geomorphologic structure of the landscapes^5^. Several studies have been carried out exploring environmental dust and its emission in the atmosphere. Many factors can influence the rates of dust emission and accumulation on surfaces. The presence of rivers, lakes, rainfall regularities, and forestry lowers dust emission and provides localized dust control^6^. Depending on the local region, dust in the atmosphere has a high concentration of inorganic compounds, such as gypsum and SiO_2_ (plaster dust)^7^. However, alkaline and alkaline earth metal compounds (NaCl, KCl, and CaCl_2_) are also reported to be present in the dust particles for those collected in close region of seashores^8^. Moreover, dust storms contribute to dust settlement on surfaces around the Globe. Although the percentage of dust settlement on surface changes regionally, the contribution of dust storms to overall dust settlement reaches almost 7.3% in northern China^9^. In addition, dust storms increase the dust particle size in the dust settlement layer, which is reported to be within the range of 9–26.1 µm^9^. However, in the Gulf region, the average dust particle size is about 1.2 µm^10^. The removal of large size dust particles from surfaces remains easier than those of the small size dust particles via utilizing the gravitational influence^4^. This is because of the dust particle shapes and texture characteristics of the dust particle surface. Large dust particles, in general, become more round shape^8^, and air trapped within the texture of the dust particle surface lowers the particle contact on the settled solid surface. This minimizes dust adhesion (due to low interfacial forces) and friction due to the small size of surface contact area between dust and dust settled surface. Hence, the gravitational force enables to initiate the motion of large size particles on inclined surfaces^4^. Moreover, the cost-effective dust removal from surfaces remains a challenge, particularly for renewable energy applications^11^. Sustainable operation and performance of photovoltaic panels are significantly influenced by environmental dust settlement^12^, even though the intensity of solar radiation reaching on photovoltaic panel surfaces is high^13^. In addition, environmental dust settlement on concentrated solar power collectors has a detrimental effect on thermal energy yield losses. In some cases, this loss increases by almost 1% daily^14^. Dust influence on optical characteristics, such as transmittance and absorption, of solar energy converting devices, is also reported to be significant, especially as the dust particles agglomerate on surfaces^15^. The mechanism of dust soiling in the near-desert area is mostly governed by airflow and the number of suspended particles in air^16^. In addition, the development of anti-soiling coating is one of the current research topics in recent years, particularly for solar energy applications^17^. The coating with anti-soiling and high optical transparency with multi-scale features are mainly required for practical applications^18^.

On the other hand, liquid droplet cleaning of dusty surfaces, mimicking nature, can be considered as one of the self-cleaning methods. For droplets to be mobile on surfaces, the Cassie and Baxter wetting state need to be satisfied, which in turn requires a hydrophobic wetting characteristic on the surface^19^. Several parameters influence the liquid droplet rolling on the dusty hydrophobic surface, such as droplet contact angle and hysteresis, droplet size, surface inclination angle, dust layer thickness, etc. In any case, some drawbacks can occur on the droplet path where the dust particles picked up by the rolling droplet. In this case, droplet wobbling under the gravitational influence generates striation like edges along the droplet path^20^. In addition, the size of the droplet path is limited with the wetting area of the droplet, which remains always smaller than the droplet diameter. This limits the area cleaned on the inclined hydrophobic surface by a rolling droplet. Increasing droplet size enhances the wetting area of the droplet fluid on the hydrophobic surface; however, droplet wobbling/puddling remains big and the size of the striations becomes large. This results in a non-uniform droplet path on the dusty hydrophobic surface. Alternatively, introducing the gravitational effect, such as tilted hydrophobic surface, enables to remove a considerable amount of settled dust from the surfaces^4^. Enhancing the area, where dust is removed, the tilt angle of the surface needs to be increased, otherwise, the dust residues cover the large area of the surface. This occurs because of the dust pinning on the hydrophobic surfaces. In order to reduce the interfacial resistance (forces) between the dust particles and the hydrophobic surface, the dust particles can be functionalized via tetraethoxysilane (TEOS) aerogels, which in turn lowers the surface free energy^21^. However, this method is expensive and requires multi-step processes. One of the methods that modify dust adhesion, because of interfacial resistance between the dust particles and hydrophobic surfaces, can involve with modification of dust chemistry in the surface region of the dust particles. It is worth mentioning that traces amount of ionic compounds, such as (NaCl and KCl), create stronger ionic forces on the dust particle surfaces while contributing to interfacial adhesion between the dust particle and surfaces. Hence, dilute acid treatment, such as hydrofluoric acid, on dust surfaces modifies the chemical nature of the dust surface region. Care is taken to observe if any, hazardous effect(s) of treated dust with a hydrofluoric acid solution. Hence, initial tests were carried out towards treating dust with diluted hydrofluoric acid solution and findings demonstrated that main constituting compound was CaF_2_ after treatment, which is recorded as not a hazardous product according to the Occupational Safety and Health Administration (OSHA, US Department of Labor) Globally Harmonized System (GHS, HCS 2012 (29 CFR 1910.1200)) and does not contain any hazardous components with a section 302 EHS TP (Emergency Planning Extremely Hazardous Substance Threshold Planning Quantity, 40 CFR 355). In the present study, the physical and chemical characteristics of the dust particles before and after dilute hydrofluoric acid treatment (solution treatment) are investigated. The adhesion work resulted during the removal of untreated and solution treated dust particles from the hydrophilic and hydrophobic surfaces is evaluated. In addition, mitigation of untreated and solution treated dust from the hydrophilic and hydrophobic surfaces under the gravitational influence is presented for various surface inclination angles. The outdoor tests are also conducted assessing the surface area at which dust removed from glass surfaces under the gravitational influence.

## Experimental

Glass sheets were prepared in 30 × 60 × 1 mm^3^ (width × length × thickness). Some sample surfaces were hydrophobized via coating by the functionalized nano-sized silica particles through adopting the dip coating method. The silica nanoparticles were synthesized in line with the early work^22^. The mixture of tetraethoxysilane (TEOS), octyltriethoxysilane (OTES), ethanol, and ammonium hydroxide solution was utilized in synthesizing cycle. In the process, 14.4 mL of ethanol, 1 mL of distilled water, and 25 mL of ammonium hydroxide were mixed and stirred for 25 min. TEOS (1 mL) was first diluted using ethanol (4 mL) and later added to the mixture and mechanically stirred for 25 min. Silane was included in the resulting mixture with a molar ratio of 3:4 and the mixture was further stirred for 15 h at room temperature. The sample surfaces were coated with the resulting mixture via the dip coating method. The reactant residuals were removed via centrifuging and the samples were finally washed with ethanol for 5 min. The roughness of the resulting coating surface was analyzed using an atomic force microscope (NanoSurf, CoreAFM) and the average surface roughness resulted was about 150 nm. The surface free energy of the coating was evaluated incorporating the droplet technique via using water, glycerol, and ethylene glycol as fluids^23^. The surface free energy of the hydrophobized surface is estimated as 35.51 mJ/m^2^. A goniometer (Kyowa, model DM 501) was used and droplet contact angle measurements were carried out in accordance with the previous study^24^. The water droplet contact angle of the coating surface is 150° ± 2° and contact angle hysteresis is 3° ± 2°.

Environmental dust was collected from photovoltaic panel surfaces while utilizing soft brushes and the collected dust was kept in air-tight containers. Scanning electron microscope (SEM, JEOL 6460), energy dispersive spectroscopy (EDS, JEOL 6460), and X-ray diffraction (Bruker D8) were used. Hydrofluoric acid was mixed with water to form the mixture solution at various concentrations (by volume). Dust was added into the mixture solution and kept for 30 min. The solution treated dust particles were dried in an open atmosphere and their geometric features were examined under SEM. The mixture solution concentration was selected on the base of the dust surface texture, i.e. the minimum solution concentration resulting in submicron/nanopores and pillars on the dust particle surface is selected. This arrangement results in 30% hydrofluoric acid and 70% water mixture. X-ray photoelectron spectroscopy (Thermo Scientific, Escalab 250Xi) was utilized for solution treated dust composition analysis. In XPS analysis, an X-ray source of Al Kα (1,486.6 eV) was used while operating at a resolution of 0.5 eV and having a 650 µm X-ray beam and pass energy of 100 eV for the survey scan, and 30 eV for the high-resolution elemental analysis. Depth profile XPS study was performed by etching the surface by Ar ion beam for about 10 s repeatedly, and XPS spectra were taken after every etching cycle. Binding energies for the high-resolution spectra were calibrated by setting C 1 s to 284.8 eV. In addition, to evaluate the dissolution of dust compounds in distilled water and the mixture solution, solution treated and collected dust particles were mixed individually with distilled water. The mixture of liquid-dust particles was filtered and the resulting solution was examined using a quadrupole inductively coupled plasma mass spectrometer (Thermo Scientific, XSeries 2). Pellets are formed from the collected and solution treated dust particles and Fourier transforms infrared spectroscopy (FTIR, Thermo Fisher Scientific, Nicolet iS50) was carried out.

## Results and discussion

Several methods have been introduced for cleaning surfaces from dust and most of these methods require external power sources, such as compressed air^25^, mechanical brushing^26^, water pump^27^, etc. In addition, self-cleaning of surfaces via water droplet rolling/sliding still requires external power for removing dust from surfaces; however, external power required for self-cleaning remains less than those of other cleaning processes. Texture characteristics of the surface remain critical for the self-cleaning application of surfaces such that surface texture resulting in a hydrophobic wetting state becomes necessary. To achieve the hydrophobic wetting state, the surface must have a texture feature consisting of hierarchically distributed micro/nanopillars. In addition, the Lotus effect needs to be created on the textured surface to increase liquid droplet mobility, i.e. enhancing rolling/sliding while reducing droplet pinning on textured surfaces, which requires creating nano-whiskers like structures on the textured surface. Generating such texture characteristics on surfaces becomes difficult and the texturing process involves ether multi-steps chemical treatments^28^ or laser ablation with high precision of surfaces^29^. In addition, a self-cleaning method involving a water droplet rolling on hydrophobic surface necessitates clean water resources for droplet formation. This becomes a difficult task in the region where water scarcity is present. Hence, environmental dust treatment is considered towards removal from hydrophilic and hydrophobic glass surfaces. The chemical composition and surface free energy of collected dust are modified via dilute hydrofluoric acid solution treatment. Adhesion of solution treated and untreated dust particles on the hydrophobic surface are examined and removal of solution treated and untreated dust from inclined hydrophilic and hydrophobic surfaces under the gravitational pull is explored.

### Untreated and solution treated dust characteristics

**Figure 1:**
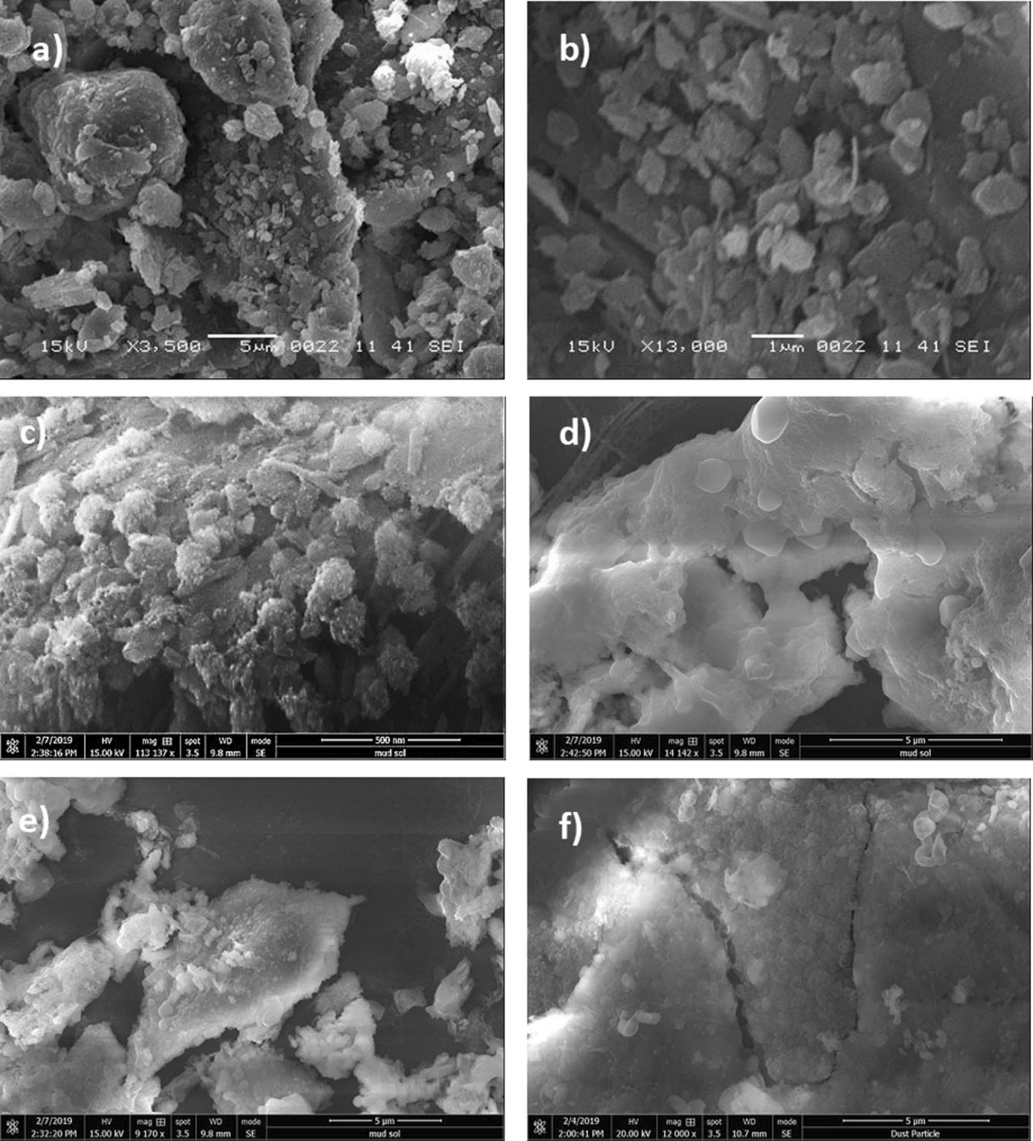
SEM micrographs of collected and solution treated dusts: (**a**) collected showing various sizes, (**b**) collected dust clusters. (**c**) Solution treated dust with submicron/nano pores and pillars, (**d**) fine size pores in solution treated dust, (**e**) mechanically anchored solution treated dust, and (**f**) micro-cracks on solution treated dust surface.

**Figure 2:**
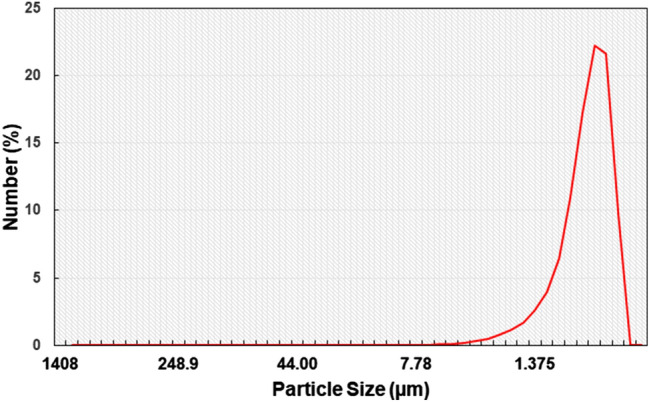
Dust particle size distribution.

**Figure 3:**
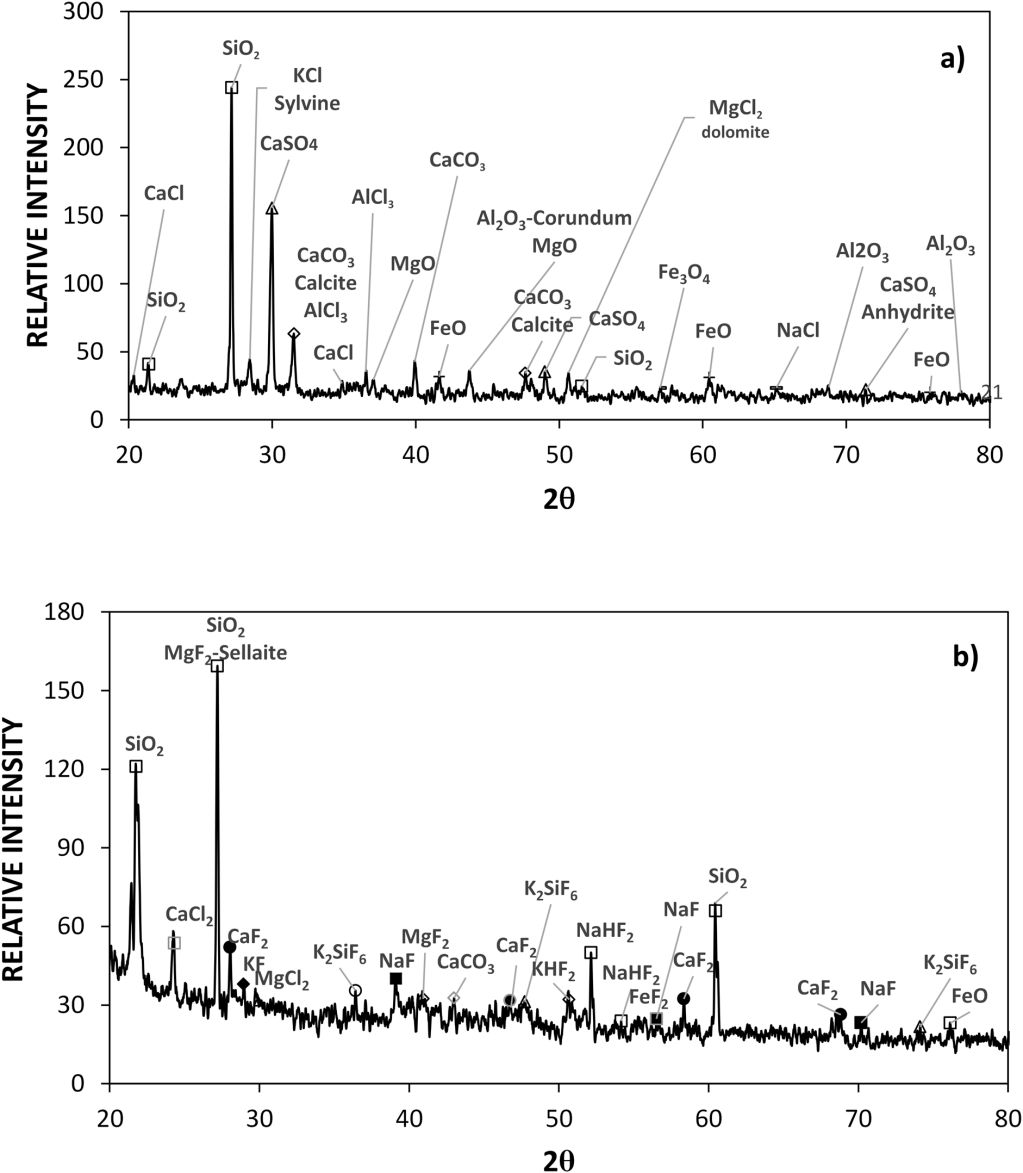
X-ray diffractogram of collected and solution treated dusts: (**a**) collected dust, and (**b**) HF treated dust.

Figure 1a and b show SEM images of collected dust (untreated dust) while Fig. 1c–f depict SEM images of the solution treated dust. Dust composes of different shapes and sizes (Fig. 1a) and some dust agglomerates forming dust clusters. The size distribution of dust particles is analyzed using particle size analyzer (Malvern Panalytical). Figure 2 shows the dust particle size distribution. The average dust particle size is about 1.2 µm. Figure 3a and b show X-ray diffractogram of collected and solution treated dust. In the case of collected dust, the sulfur peak occurs because of the calcium in the form of anhydrite or gypsum (CaSO_4_). The iron peak is due to clay-aggregated hematite (Fe_2_O_3_). Some dust compounds are formed from alkaline (Na, K) metals with chlorine, which are attributed to the salt compounds in the dust. Table 1 gives the elemental composition of collected dust. It is worth noting that in the assessment of the elemental constitutes through EDS analysis, the dust particles are grouped into two size categories, which include the particles smaller and equal to the average size dust particles (≤ 1.2 µm) and those larger than the average size. Since SEM micrographs are used in EDS quantification of the elemental composition, this allows grouping of the dust particles in sizes. In addition, EDS analysis is repeated for several sizes of the dust particles within the size groups and findings demonstrate that variation of the quantified data for each dust particle size group does not change notably, i.e. the change is less than 1%. The elemental composition of collected dust slightly changes as the dust particle size reduces (≤ 1.2 µm), i.e. oxygen content in the dust compounds increases for small dust particles. Nevertheless, the dust has various elements including Fe, Si, Ca, K, Na, S, O, and Cl. EDS data (Table 1) reveals that some compounds do not satisfy the stoichiometric elemental ratios such as Na_x_Cl_y_ and K_m_Cl_n_, where x ≠ y and m ≠ n. The complexity of the compounds potentially creates strong ionic forces on the dust particles. Hence, these forces contribute to the adhesion of dust particles while forming the dust clusters (Fig. 1b). In the case of dilute hydrofluoric acid-treated (solution treated) dust (Fig. 1c–f), the surface modifies toward more fluorinated and hydrophobic surfaces. In addition, the solution treated dust particle surfaces possess pores and submicron/nanopillars like structures (Fig. 1c) because of dissolution of some dust compounds in the acidic solution during the dust treatment. However, as collected dust particles mixed with desalinated water, the pH of the mixture increases from 5.8 to 8.6 after five minutes. This shows that the surface of the collected dust is rich in dissolved alkaline (Na, K) and alkaline earth metal (Ca) compounds which increased the pH. As the dust particles are mixed with the hydrofluoric acid solution (30% hydrofluoric acid and 70% water), several reactions can take place between hydrofluoric acid solution and dust. Some compounds of the dust particles (CaCO_3_, NaCl, SiO_2_, etc.) in the mixture solution undergo reactions with hydrofluoric acid. Some of these reactions can include:

**Table 1:** Elemental composition of dust particles (wt%).

	Size	Si	Ca	Na	S	Mg	K	Fe	Cl	F	O
Collected	≥ 1.2 μm	11.8	8.3	2.2	1.3	2.5	0.8	1.2	0.4	0	Balance
Collected	< 1.2 μm	10.2	7.3	2.7	2.5	1.3	1.2	1.1	1.1	0	Balance
Solution treated	≥ 1.2 μm	8.1	8.2	1.2	1.1	1.8	0.5	0.8	0.2	27	Balance
Solution treated	< 1.2 μm	7.6	7.4	1.3	1,2	0.9	0.7	0.7	0.6	31	Balance

1 $$Si{O}_{2}+6HF\to {H}_{2 }Si{F}_{6}+2{H}_{2}O$$

Hence, hexafluorosilicate (${H}_{2 }Si{F}_{6}$) can be formed. Since, salt compounds (NaCl, KCl) is present in dust, this can undergo reaction with hexafluorosilicate, i.e.:

2 $$2NaCl+{H}_{2 }Si{F}_{6}\to {Na}_{2}Si{F}_{6}+2HCl$$

Similarly, KCl undergoes a reaction while forming:

3 $$2KCl+{H}_{2 }Si{F}_{6}\to {K}_{2}Si{F}_{6}+2HCl$$

${Na}_{2}Si{F}_{6}$ and ${K}_{2}Si{F}_{6}$ are the crystalline solids, which can dissolve in a mixture solution and it is denser than water. However, the presence of calcite (CaCO_3_) in dust can also undergo a reaction with HCl, which is produced after the NaCl reaction with hexafluorosilicate (${H}_{2 }Si{F}_{6}$), i.e.:

4 $$CaC{O}_{3}+2HCl\to Ca{Cl}_{2}+C{O}_{2}+{H}_{2}O$$

*CaCl*_*2*_ is highly soluble salt in water and dissolves in the mixture solution. In addition,

5 $$CaC{O}_{3}+2HF\to Ca{F}_{2}+C{O}_{2}+{H}_{2}O$$

Similarly, magnesium oxide (MgO) in dust can react with the mixture solution, i.e.:

6 $$MgO+2HF\to Mg{F}_{2}+{H}_{2}O$$

and with HCl:

7 $$MgO+2HCl\to Mg{Cl}_{2}+{H}_{2}O$$

**Figure 4:**
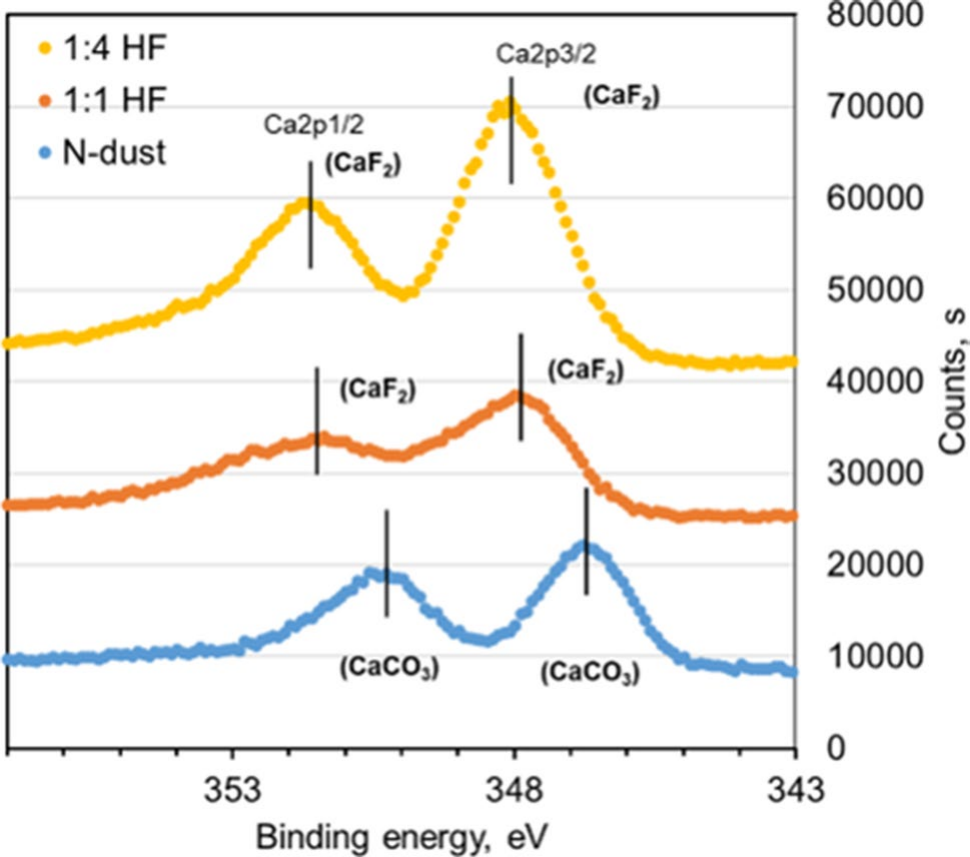
XPS data for collected and solution treated dusts. HF represents hydrofluoric acid at two different concentration. 1/4 HF represents 25% HF concentration in dust, 1:1 HF represents 50% HF concentration in water, N represents collected dust. In all cases, HF is diluted in 70% water.

**Figure 5:**
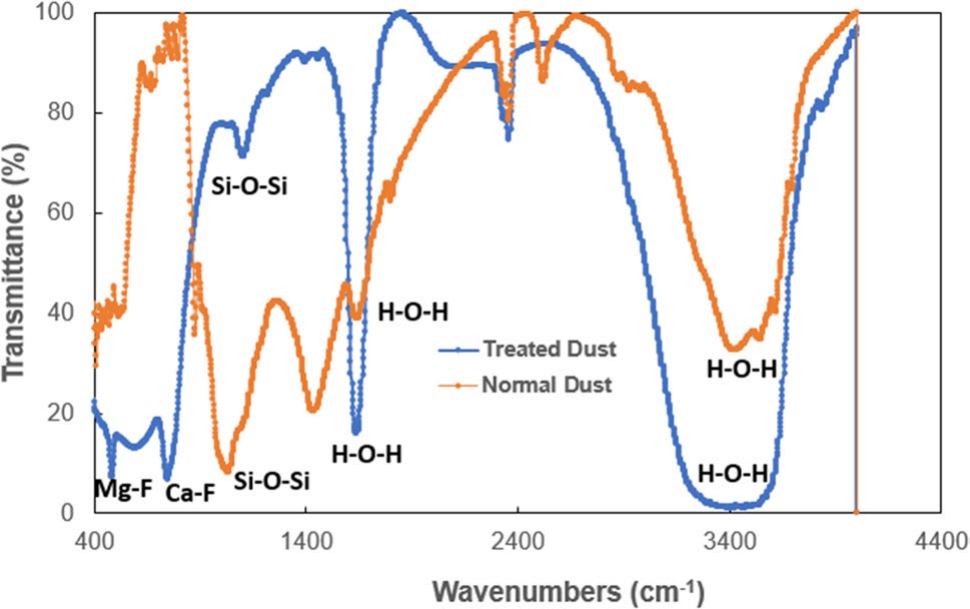
FTIR data for untreated and solution treated dusts.

**Figure 6:**
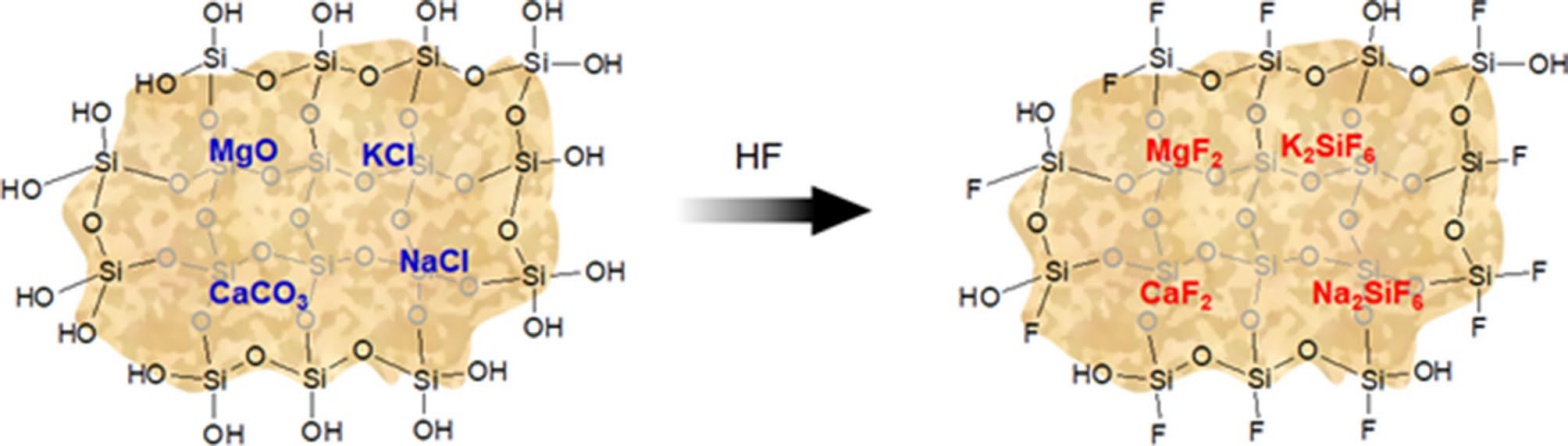
Schematic diagram of collected dust particle treatment with concentrated HF.

MgF_2_ is the fluorescent crystals, which cannot dissolve in water; however, MgCl_2_ is the magnesium salt, which can dissolve in water. Table 1 gives the elemental composition of dust after solution treatment. The elemental composition of treated dust differs from untreated dust (Table 1). The main differences are associated with the reduction of quantification of some elements and the addition of the presence of fluorine in Table 1. Several new compounds are formed for solution treated dust, which can be observed from the x-ray diffractogram (Fig. 3b). The peaks of $Ca{F}_{2}$, $Mg{F}_{2}$, ${Na}_{2}Si{F}_{6}$, and ${K}_{2}Si{F}_{6}$ demonstrate the solution treated dust possess new fluorine compounds. Further characterization analyses are carried out for the solution treated dust particles and characteristics of solution treated dust were examined using X-ray photoelectron spectroscopy (XPS). Table 2 summarizes the elemental composition of samples estimated XPS analysis. Solution treated dust shows the increased composition of fluorine and calcium, and diminished composition of oxygen and silicon. This demonstrate that the surface of solution treated dust have CaF_2_ content as reference to the collected dust. The details of high-resolution XPS analysis (as shown in Fig. 4) reveal on shifting of Ca2p peak from 347 eV, which is characteristic to CaCO_3_ to 348 eV characteristic of CaF_2_ for the solution treated dust. Moreover, Fig. 5 shows Fourier infrared spectroscopy (FTIR) data for solution treated dust. The peak occurring at 1,128 cm^−1^ corresponds to Si–O–Si rotational vibration as described in the early work^30^. This demonstrates that polysiloxane compound is formed on Na_2_SiF_6_ surface^31^. The peak at 477 cm^−1^ is due to stretching vibration of Mg-F^32^. In addition, peak at 3,448 cm^−1^ and 1661 cm^−1^ are the characteristic bending vibration of hydroxyl groups (H–O–H)^33^. The peak observed at 775 cm^−1^ is attributed to Ca-F stretching vibration^34^. The chemical reaction occurring with the dust compounds around the dust particles almost eliminates the adhesion of small size dust particles on the dust particle surface, i.e. clustering of the dust particles diminishes as observed for collected dust, i.e. individually oriented dust particles are observed on the surface. In addition, solution treatment results in some salt products, such as ${Na}_{2}Si{F}_{6}$, ${K}_{2}Si{F}_{6}$ and $Ca{Cl}_{2}$, and $Mg{Cl}_{2}$, which are the inorganic compounds and they can dissolve in the liquid mixture. Overall, the transformation of collected dust particles upon HF treatment can be pictorially summarized as shown in Fig. 6.

**Table 2:** XPS data for collected and solution treated dust.

	O1s	C1s	Si2p	Ca2p	N1s	Al2p	F1s
**Prior etching**							
Collected	52.11	23.19	10.82	5.17	0.27	5.71	1.10
Solution treated	30.19	21.93	3.82	10.04	1.41	2.61	29.99
**After 20 s etching**							
Collected	58.26	9.15	12.75	7.14	1.03	7.42	0.00
Solution treated	26.86	8.72	7.60	11.99	0.00	3.42	41.32

The solution treated dust particles are mixed with desalinated water for 1 h and, later, the resulting solution is tested by inductively coupled plasma mass spectrometer (ICP) for Ca, Mg, K, and Na. In addition, the same ICP tests are also repeated for the collected dust particles. Table 3 gives ICP data for collected and solution treated dust particles. The amount of dissolved calcium in water reduces for the solution treated dust, which is attributed to the formation of CaF_2_ and CaCl_2_, i.e. only CaCl_2_ is soluble in water. The amount of dissolved magnesium and potassium attain larger values for solution treated dust as compared to the collected dust. The compound of ${\mathrm{MgCl}}_{2}$ salt is soluble in water, which increases Mg content in the solution. Sodium reduces slightly for the solution treated dust as compared to that of the collected dust. Moreover, the dissolution of the products around the dust particle results in the formation of local porous structures on the dust particle surface (Fig. 1d). Since the elemental distribution of alkaline salt compounds within the dust particle is nonuniform, this causes irregular sub-micro/nano pores and pillars like structures around the dust particles. Hence, irregular texture patterns are observed on the treated dust particle surface (Fig. 1c). The irregular texture patterns with sub-micro/nano pores and pillars on the dust surface gives rise to interlocking of the dust particles when the particles are mechanically in contact to each other. Hence, mechanical locking of the particles after solution treatment is observed (Fig. 1e). As the solution treated particles are located on the smooth surface, the area of contact between the dust particle and the smooth surface becomes less because of the fact that the interfacial gaps, due to submicron/nano pores and pillars, are filled with an air. This lowers the adhesion between the solution treated dust particles and the smooth surface. CaF_2_ compound formed around the dust particle is insoluble solid in water and remains in the surface region of the dust particles. The density of CaF_2_ (3,180 kg/m^3^), which is formed locally around the dust particle, differs than the density of dust (2,800 kg/m^3^). This can cause formation of mechanical strains, due to volume contraction/expansion, and as a result some small local cracks are formed on the dust surface (Fig. 1f). However, the size of cracks are small and do not form crack-webs on the dust surface.

**Table 3:** ICP data for collected and solution treated dusts.

	Ca 317.933 (mg/L)	K 766.490 (mg/L)	Mg 285.213 (mg/L)	Na 589.592 (mg/L)
Collected	78.17	8.118	8.581	15.77
Solution treated	37.58	56.31	144.2	11.79

### Surface free energy assessment and liquid spreading

The surface free energy of collected and solution treated dust is assessed incorporating the contact angle measurement technique^23^. Three liquids namely water, glycerol, and ethylene glycol are used in contact angle measurements. To measure the contact angle, two procedures are adopted. In the first approach, the Washburn technique^35^ is employed to measure the liquid contact angle on dust and in the second approach small dust pellets are formed via slight compression of the dust particles, and contact angle measurements are carried out using the Goniometer adopting the previous procedure^24^. In the case of the Washburn method, a small diameter (3 mm) glass tube is used and dust particles are put into the tube while the tube is placed on a small liquid container. Hence, the dust particles can draw-up the liquid via liquid infusion. The mass increase in the tube due to liquid infusion is related to the Washburn equation, i.e.: $\frac{{(\Delta m)}^{2}}{\Delta t}=\frac{c\cdot {\rho }^{2}\gamma cos\theta }{\mu }$, here *Δm* represents the mass gain, *Δt* is the duration for the mass gain (flow-time), *c* is the capillary constant of dust, *ρ* corresponds to the liquid density, *θ* is the contact angle, *µ* represents the liquid viscosity. The value of *c* (capillary constant) for dust is evaluated using n-hexane as a liquid, which gives rise to zero contact angle (*θ* = 0). Hence, the value of *c* is determined as 6.84 ± 1.3 × 10^−16^ m^−5^ for collected dust particles and 5.52 ± 1.2 × 10^−16^ m^−5^ for the solution treated dust particles. The variations of the capillary constant for collected and solution treated dust are related to shape and size of the dust particles since force balance generated due to capillary and gravity changes on the particle surface as the size and shape of the particle change, which is also reported for powders in the early study^36^. Nevertheless, the estimation for the capillary constant varies within less than 20%. The contact angle measured for the water using the Washburn method is 37.2° ± 3° for collecting dust and it is 38.4° ± 3° for solution treated dust. In the case of dust pellets, the contact angle measured, via goniometer, is about 38.2° ± 3° for collecting dust and it is 38.7° ± 3° for solution treated dust. Experiments are repeated seven times ensuring the repeatability of the contact angle measurements and the experimental error estimated is within 8%. Nevertheless, the Washburn method incorporated in the contact angle measurement results in a similar droplet contact angle to that obtained from the goniometer measurements on pellet surfaces. The surface free energy of dust is evaluated using the formula developed for liquid–solid contact, i.e.: ${\gamma }_{L}\left(\mathit{cos}\theta +1\right)=2\sqrt{{\gamma }_{S}^{L}.{\gamma }_{L}^{L}}+2\sqrt{{\gamma }_{S}^{+}.{\gamma }_{L}^{-}}+2\sqrt{{\gamma }_{S}^{-}.{\gamma }_{L}^{+}}$^23^. The subscripts *L* and *S* are for liquid and solid phases, *γ*_*S*_ is the surface energy for solid, *γ*_*S-L*_ represents free energy for the solid–liquid interface, *γ*_*L*_ is liquid surface tension, *θ* corresponds to contact angle, γ^+^ and γ^−^ are parameters for electron acceptor and donor due to the acid–base component of free energy for solid and liquid phases. Table 4 gives the data for Lifshitz-van der Walls components and electron-donor parameters^23,37^. The surface energy estimated from the contact angle measurement method for collected dust is around 112.3 ± 7.8 mJ/m^2^ while it is about 94.8 ± 6.2 mJ/m^2^ for solution treated dust. Since several new compounds are formed in the dust surface region after the treatment, such as *CaF*_*2*_ and some salt compounds (${Na}_{2}Si{F}_{6}$, ${K}_{2}Si{F}_{6}$ , CaCl_2_ and MgCl_2_) the surface free energy of collected dust changes after the solution treatment. It should be noted that the interfacial chemical feature of the compounds, particularly fluorite compounds play an important role on wetting state of the liquid on the surface^38^, e.g. the cleavage plane of *CaF*_*2*_ (111) results in low surface energy than *CaF*_*2*_ crystals with different structure, such as (100) and (110) planes. Moreover, the spreading rate of the liquid on dust depends on the spreading coefficient (*S*). The spreading factor for liquid infusing on a solid surface satisfies the relation: $S={\gamma }_{s}-{\gamma }_{L}-{\gamma }_{s-L}$^39,40^. Here, *γ*_*s*_ represents surface free energy of dust, *γ*_*L*_ represents liquid surface tension, and *γ*_*s–L*_ is interfacial tension of liquid-dust pair. The interfacial tension can be evaluated via ${\gamma }_{s-L}={\gamma }_{s}-\frac{{\gamma }_{L}}{r}\mathrm{cos}\theta$^40^. Here, *r* is the roughness parameter, which represents the ratio of the area of pillars in the texture over the projected area of the textured surface, and it takes the values between 1 and 0; here, 1 being the extremely rough surface and 0 being the extremely smooth surface. After close examination of several SEM micro-images of the dust particles, the roughness parameter is evaluated as *r* = 0.52 for collecting dust and *r* = 0.62 for solution treated dust. Since the water contact angle is 37.2° ± 3° for normal dust and it is 38.4° ± 3° for solution treated dust and substituting surface energies obtained from contact angle methods, the interfacial tension between dust and water is estimated to be ${\gamma }_{s-L}\hspace{0.17em}=\hspace{0.17em}$2.1 mJ/m^2^ for collecting dust and it becomes ${\gamma }_{s-L}=$ 3.74 mJ/m^2^ for solution treated dust. The initial period of liquid spreading over the dust particle is governed by liquid infusion (cloaking), which takes place via forming a thin layer of liquid onto the dust particles in line with the Joos, law^41^ and energy dissipated by the liquid during infusion is associated with the Ohnesorge number ($Oh=\mu /\sqrt{\rho a{\gamma }_{L}}$), where *a* is the size of the dust particle^42^. Incorporating the dust particle size between 20 and 0.5 µm, Ohnesorge number takes the values within the range of ~ 0.025–0.105; hence, dissipated energy of the liquid during cloaking becomes small, i.e. liquid totally covers the dust particles. This argument is true for the solution treated and collected dust particles, which are also observed during the experimental tests.

**Table 4:** Lifshitz–van der Walls components and electron-donor parameters used in the simulation^24,37,45^.

	γ_L_ (mJ/m^2^)	${\gamma }_{L}^{L}$ (mJ/m^2^)	${\gamma }_{L}^{+}$ (mJ/m^2^)	${\gamma }_{L}^{-}$ (mJ/m^2^)
Water	72.8	21.80	25.5	25.5
Glycerol	63.3	33.11	10.74	21.23
Ethylene glycol	48.2	31.09	6.59	11.16

### Dust particles adhesion and dust mitigation

**Figure 7:**
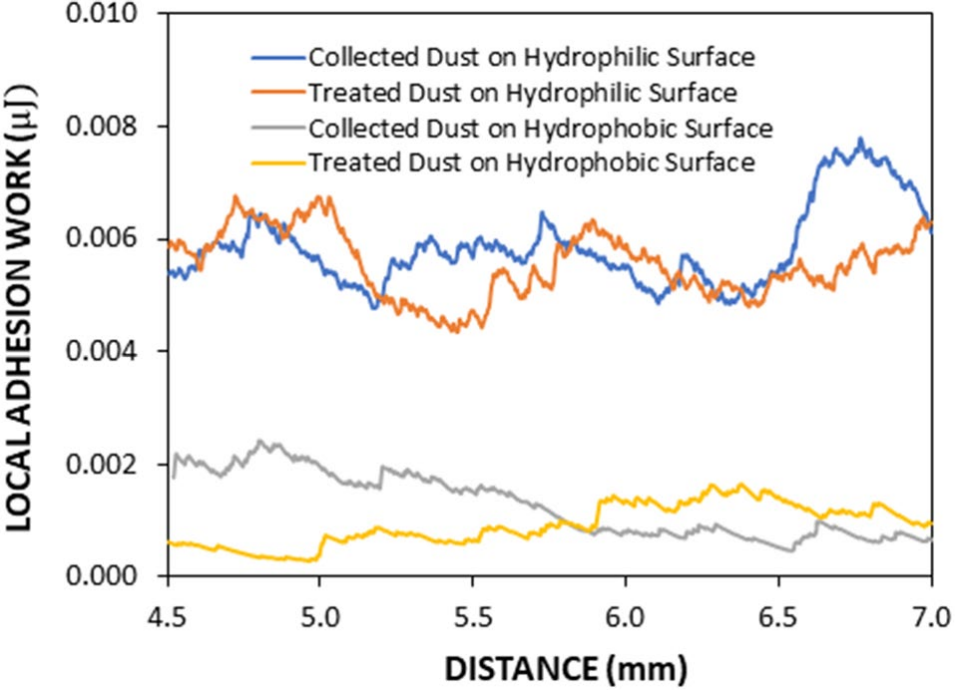
Local adhesion work along the scanning distance on dusty sample surfaces. Local adhesion work represents the adhesion work over the incremental distance where the probe scans on the surface (${W}_{ad-\Delta l}={F}_{cor}\times \Delta l$, where $\Delta l$ is the scratch tester probe minimum incremental length on the dusty sample surface). Figure is produced by using Excel 2020.

**Figure 8:**
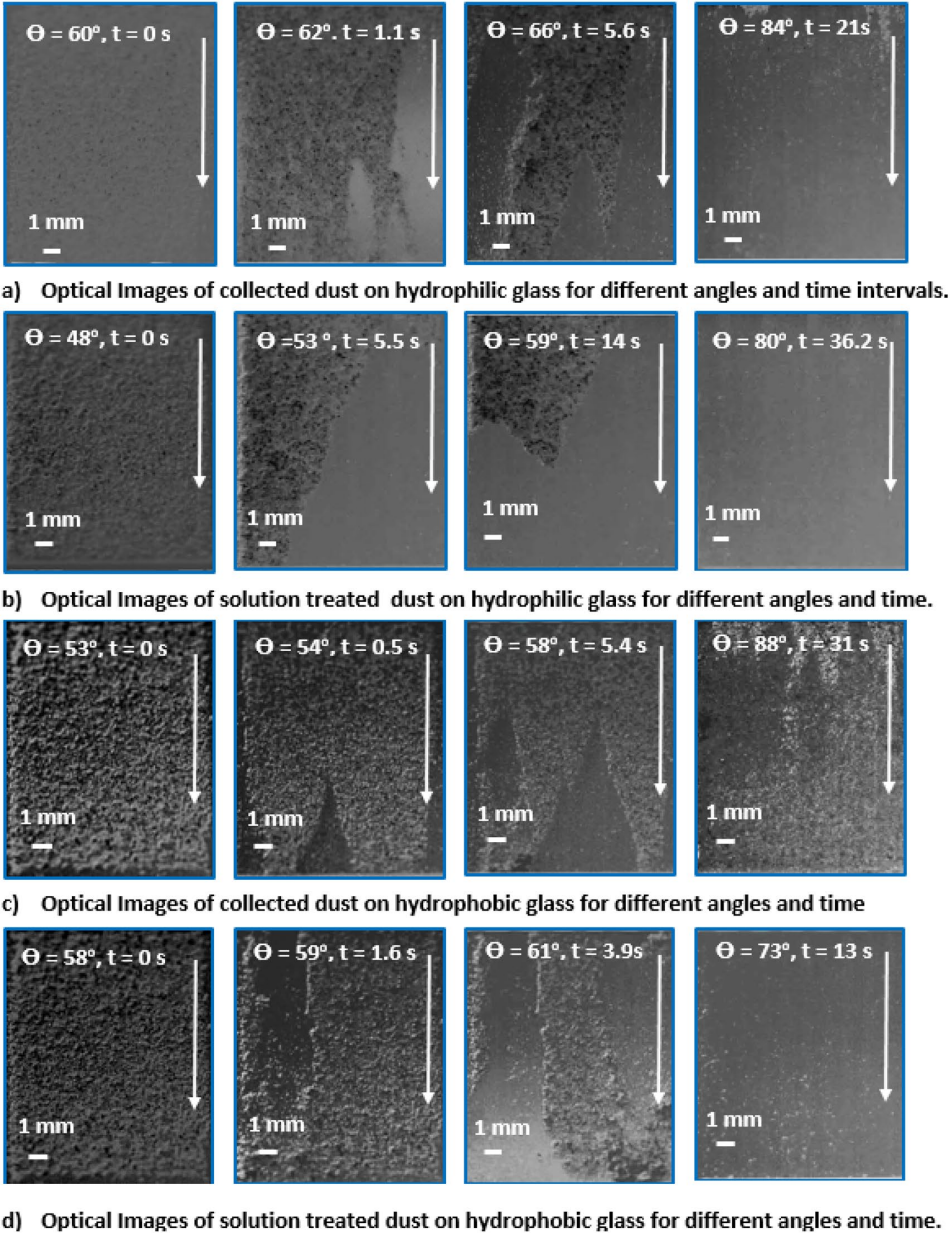
Optical images of dusty sample surfaces at different titling angle and corresponding tilting time.

**Figure 9:**
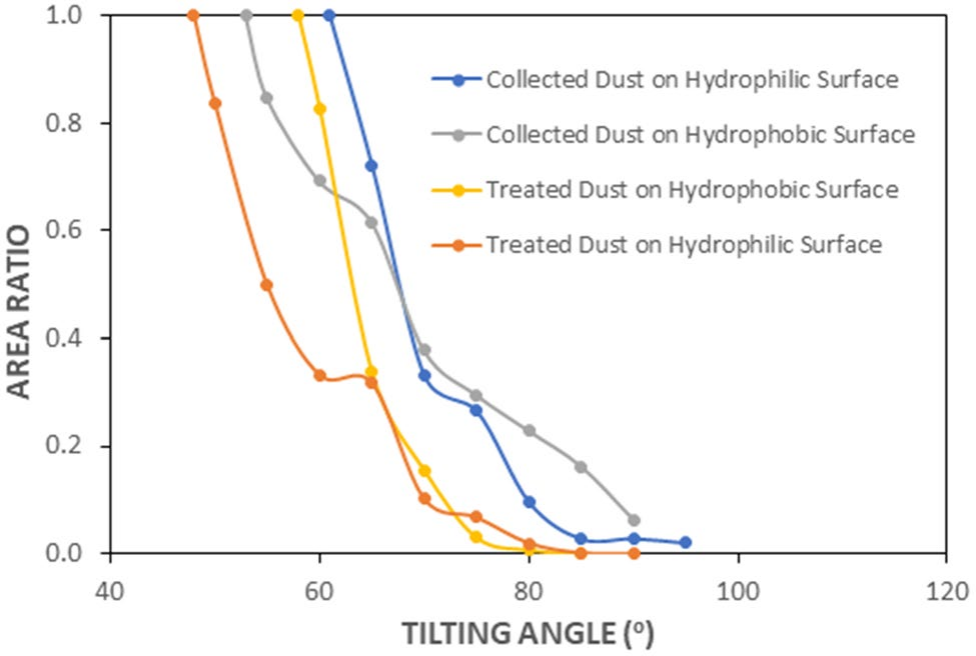
Area ratio with tilting angle of sample surface. Area ratio (${\eta }_{A}$) represents area of dust removed over total area of the dusty surface prior rotation is estimated, i.e. ${\eta }_{A}=\frac{Dusty\, Surface\, Area }{Total\, Surface\, Area}$. Figure is produced by using Excel 2020.

**Figure 10:**
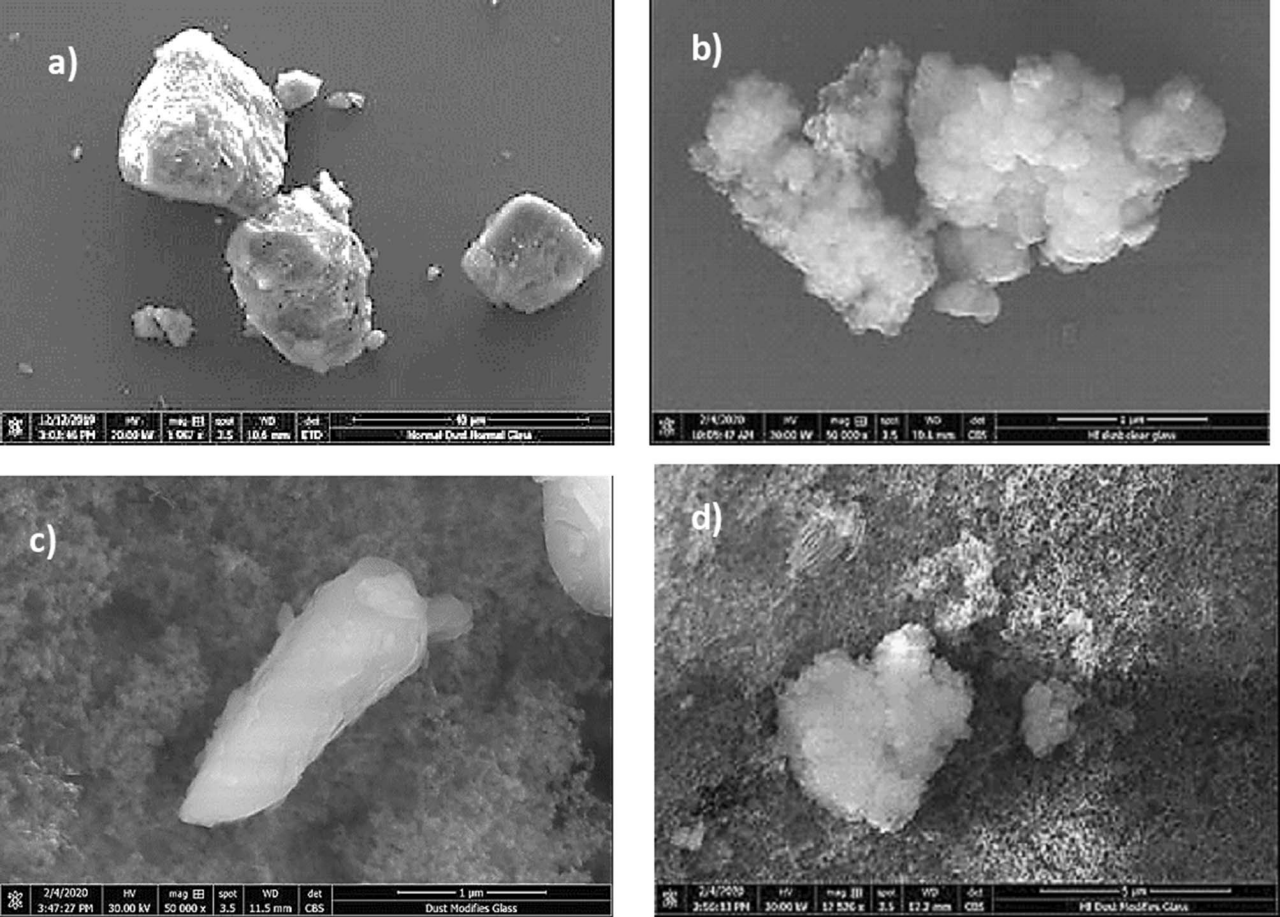
SEM micrographs of dust residues on glass surfaces: (**a**) collected dust on hydrophilic surface, (**b**) solution treated dust on hydrophilic surface, (**c**) collected dust on hydrophobic surface, and (**d**) solution treated dust on hydrophobic surface.

The hydrophobic surfaces have texture characteristics consist of hierarchically distributed micro/nanopillars, which reduces the particle pinning due to adhesion on surfaces^43^. The tests are carried out to assess the adhesion of solution treated and collected dust particles individually on the hydrophobic and hydrophilic glass samples using the atomic force microscopy (AFM); at which, the force of AFM probe defection is associated with the formula $F=k{\sigma }_{d}\Delta V$, where *k* is the probe tip spring constant (N/m), *σ*_*d*_ is the slope of the deflection at friction mode (*Δz*/*ΔV*, m/V), and *ΔV* represents the voltage (mV) recorded during AFM probe deflection^44^. The AFM probe used has $k{\sigma }_{d}\hspace{0.17em}$= 5.80275 × 10^−13^ N/mV and the probe voltage recorded for 1.2 µm collected dust particle on the hydrophilic glass sample surface is 290 mV, which results in adhesion force of about 1.68 × 10^−10^ N. However, the probe voltage recorded for the same size collected dust particle on the hydrophobic glass surface is about 90 mV, which corresponds to the force of adhesion of 0.52210^−10^ N. Hence, hydrophobic surface lowers the dust particle adhesion almost 1/3 of that of the hydrophilic surface. Experiments are repeated for the assessment of adhesion force for the solution treated dust particles on the hydrophilic and hydrophobic glass surfaces. The probe voltage recorded for about 1.2 µm solution treated dust particle on the hydrophilic glass sample surface is 210 mV and it results in the adhesion force of 1.22 × 10^−10^ N, which is almost 70% of that of the collected dust. Hence, a low concentration of hydrofluoric acid solution treated dust particles results in low adhesion to the hydrophilic glass surface as compared to that of the collected dust particle. This behavior is attributed to the surface texture characteristics of the solution treated dust, i.e. some dust compounds formed during the solution treatment (Na_2_SiF_6_, K_2_SiF_6_, CaCl_2_, and MgCl_2_) can dissolve in the solution while creating submicron/nano pores textures on the solution dust particle surface (Fig. 1c). This reduces the area of contact between the solution treated dust particle and the glass surfaces. In the case of similar size of the solution treated dust particle, the AFM probe reading is about 65 mV and it gives rise to the adhesion force of 0.38 × 10^−10^ N, which is about 75% of that of the collected dust on the hydrophobic surface. This indicates that solution treatment of dust lowers the adhesion force of the dust particle on the hydrophobic surface as compared to that of the collected dust particle. Furthermore, the adhesion tests are conducted determining the average adhesion work required to remove the solution treated and collected dust particles from the hydrophobic and hydrophilic sample surfaces. The solution treated and collected dusts with same quantity are located on the glass sample surfaces and micro-scratch tester (CSM Instruments, Micro Scratch Tester (MST)) was used to record the resistance force and corresponding probe scanning distance along the scanning line on the glass sample surface. In the tests, the hydrophobic and hydrophilic samples are used separately. To assess the correct adhesion work for the solution treated and collected dusts, the resistance force due to friction on the sample surfaces are initially evaluated using micro-scratch tester. To obtain the corrected tangential force (*F*_*cor*_), the tangential force measured along the probe path (*L*_*d*_, total dust removal length along the scanning direction) is subtracted by the frictional force along the same length. It is worth to mention that the local adhesion work is recorded, which represents the adhesion work over the incremental distance where the probe scans on the surface (${W}_{ad-\Delta l}={F}_{cor}\times \Delta l$, where $\Delta l$ is the scratch tester probe minimum incremental length on the dusty sample surface). Figure 7 shows the local adhesion work along the probe path (*L*_*d*_) for collected and solution treated dusts on the hydrophilic and hydrophobic sample surfaces. The local adhesion work becomes smaller for the solution treated dust than that corresponding to collected dust. This situation is more apparent for the hydrophobic sample surface. Hence, the solution treatment of the dust particles not only lowers the surface energy, but reduces the dust particle adhesion on the surface, despite the fact that submicron/nano textures are formed on the solution treated dust particles, i.e. these textures can act as anchoring sites towards pinning the dust particles on the sample surface. Since, the texture height of the solution treated surface is considerably small, texture influence on pinning becomes negligibly small. On contrary, the surface texture of the solution treated dust reduces the contact area between the dust particle and the sample surface while suppressing the dust pinning force on the sample surface. In addition, hydrophobic texture results in lower tangential force required to remove the dust particles from the surface. Hence, hydrophobizing the surface through generating nano-size texture with low surface energy coating on the samples reduces the dust pinning force considerably, which becomes more apparent for the solution treated dust. The average work of adhesion (*W*_*ad*_) is determined through numerical integration of local adhesion work (${W}_{ad-\Delta L}$) over the total length of probe scan (*L*_*d*_, which is also the length of dust removed by the probe on the sample surface during scanning) i.e. ${W}_{ad}=\frac{1}{{L}_{d}}{\int }_{0}^{{L}_{d}}{W}_{ad-\Delta L}dl$, here *dl* is the length scale variable. Hence, the average work of adhesion is about 4.9758 µJ for collected dust while it is 0.9361 µJ on the hydrophobic glass surface. The average adhesion work (*W*_*ad*_) for solution treated dust on the hydrophilic glass surface it is 4.0912 µJ while it is 0.6027 µJ on the hydrophobic sample surface. Hence, solution treatment of dust lowers the average adhesion work on the sample surfaces, which is more pronounced for the hydrophobic surfaces. Furthermore, outdoor tests are carried out to assess the adhesion of the collected and solution treated dust on the inclined hydrophobic and hydrophilic glass surfaces. The fixture is designed to incline the samples at 1° increments along the horizontal axis and the gravitational pull allows the dust particles slide over the sample surfaces. The tests are repeated separately for solution treated and collected dust located on the hydrophilic and hydrophobic glass surfaces. Figure 8 shows optical images of dusty surfaces of hydrophobic and hydrophilic samples after the rotation for solution treated and collected dusts. It should be noted that the rotational angle for complete removal of collected and solution treated dusts changes for the hydrophilic and hydrophobic surfaces. In this case, inclination angle for total dust removal from surface becomes smaller for solution treated dust on the hydrophobic surface as compared to those of: (1) solution treated dust on hydrophilic glass surface, (2) collected dust on hydrophobic surface, and (3) collected dust on hydrophilic surface. Hence, solution treated dust has low adhesion on the sample surface than the collected dust, which is particularly true for the hydrophobic surface. However, some dust residues are observed on the sample surfaces. In order to assess, the percentage of dust cleaned by the rotation of the dusty samples, the ratio of the area (${\eta }_{A}$) of the dust that remains over the total area of the dusty surface prior rotation is estimated, i.e. ${\eta }_{A}=\frac{Dusty\, Surface\, Area }{Total\, Surface\, Area}$. Figure 9 shows that the ratio (${\eta }_{A}$) of dusty area on the sample surface decreases with increasing inclination angle for the cases: (1) solution treated dust on hydrophobic surface, (2) solution treated dust on hydrophilic surface, (3) collected dust on hydrophobic surface, and (4) collected dust on hydrophilic surface. The inclination angle of the surface for 98% of the area ratio remains the minimum for the solution treated dust on the hydrophobic surface, then follows collected dust on hydrophobic surface, solution treated dust on hydrophilic surface, and finally collected dust on hydrophilic surface. Consequently, solution treatment of dust particles can be removed from the hydrophobic surface at low inclination angle of the surface, which reduces the mechanical work required to rotate the surface. To evaluate the dust residues shape and size, microscopic analysis is carried out. Figure 10 depicts SEM micrographs of residues of typical solution treated and collected dust particles on hydrophobic and hydrophilic surfaces. Dust residues are small in size with various shapes. In general, the dust particles having sizes > 1 µm can mitigate from the inclined surfaces under the gravitational influences. Hence, the small size dust particles remain as residues on the inclined surfaces, which is more apparent for untreated dust particles. The dust particles having sizes more than 1 µm has slightly larger texture heights and roughness parameter than those of the small particles (< 1 µm). This increases the air gap between the large size dust particles and the glass surface while lowering interfacial contact between the dust particle and the glass surface. The force of gravity acting on the large size particle is larger than the small size particles, which, enables to initiate dust must mitigation at low angle of inclination of the glass surface. In addition, small size dust residues are mainly because of the anchoring of small dust particles on the sample surfaces, which is particularly true for hydrophobic sample surfaces. Nevertheless, they are only few on the sample surfaces. Introducing hydrofluoric acid treatment lowers the surface energy of the dust particles and texture the dust surfaces while lowering contact area between the dust particles and the glass surfaces. This significantly reduces the dust adhesion and eases dust mitigation under the gravitational influence. However, the methods developed for dust mitigation such as compressed air blowing^25^ or liquid jet cleaning^27^ or droplet rolling^4^ require large facilities and clean water, which may be difficult to obtain in regions where the clean water sources are limited. Since hydrofluoric acid is ready available at low cost, adopting hydrofluoric acid treatment on dust particles can ease the additional power requirements for dust mitigation from surfaces. Moreover, using the laboratory facilities at King Fahd University of Petroleum and minerals, surface cleaning tests are conducted for the dusty glass surfaces. Initially, the dust layer of about 80 µm uniform thickness is formed on the glass surface of 1 m^2^. The dust layer is removed separately by clean water via water jet splashing and compressed air blowing via air jet nozzle. The estimate from the laboratory tests for the dust mitigation from the glass surfaces per meter square reveal that the cost of hydrofluoric acid treatment is almost half cost of the dust mitigation by the clean water splashing and almost one quarter of the air jet blowing. On the other hand, the detailed techno-economic analysis is required for proper comparison of the current method with the other available methods for dust mitigation from surfaces. Since the mechanisms and characteristics of dust treatment, via diluted solution of hydrofluoric acid, are explored in the present study, further detailed techno-economic analysis for cleaning is left for future study.

## Conclusion

Environmental dust modifications towards their removal from surfaces are considered. The solution modified and collected dust particles adhesion on hydrophilic and hydrophobic glass surfaces are examined. The dust particles are collected from the Dammam area of Saudi Arabia and a low concentration hydrofluoric acid solution (30% HF and 70% water mixture) is used to treat the dust particles. The collected and solution treated dust particles are chemically analyzed and dissolution of the components of the dust particles in water-hydrofluoric acid mixture is evaluated. The surface free energies of collected and modified dust are estimated using the droplet method. Adhesion of the dust particles on hydrophilic and hydrophobic glass surfaces is assessed using atomic force microscopy and scratch testing methods. Experiments are extended to include dust removal from inclined hydrophilic and hydrophobic glass surfaces under the gravitational influence and the resulting area of dust removed and dust residues are examined incorporating the digital imaging system. The findings demonstrate that environmental dust possesses various elements and compounds and some of these compounds do not satisfy the stoichiometric elemental ratio while causing cationic/anionic forces on the dust surface. This gives rise to the attachment of some small size dust particles forming the dust clusters. The low concentration hydrofluoric acid solution treatment of collected dust particles minimizes this effect and the dust clustering ceases. Some new complex salt compounds are formed in the dust particles during the solution treatment, which causes micro/sub micro size cracks on the dust particle surfaces. Some compounds of solution treated dust can dissolve in the solution while creating sub-micron/nanopores and pillars on the surface of the dust particles. The adhesion force obtained from AFM analysis for solution treated dust on the hydrophilic and hydrophobic glass surfaces remains almost 1/3 of that of the collected dust. The solution treatment reduces dust adhesion work on both hydrophilic and hydrophobic surfaces, which can be attributed to: (1) solution treated dust has lower surface free energy than that corresponding to collect dust while reducing interfacial molecular forces between the dust particles and the glass surface, and (2) surface texture generated on the dust particles after solution treatment lowers the mechanical contact area between the dust particles and the glass surface. The outdoor tests revealed that inclination angle of the glass surface becomes less for the solution treated dust removal from the glass surface as compared to collected environmental dust; hence, the gravitational influence cleans the glass surface from dust at low tilt angle as the dust is treated by low concentration hydrofluoric acid. The present study gives insight into environmental dust adhesion on hydrophilic and hydrophobic surfaces and introduces a useful approach for dust removal from glass surfaces via solution treatment.

## Data Availability

Test data are available on request.

## References

[1] Taheri F, Forouzani M, Yazdanpanah M, Ajili A (2020). How farmers perceive the impact of dust phenomenon on agricultural production activities: a Q-methodology study. J. Arid Environ..

[2] Soleimani Z (2019). An overview of bioaerosol load and health impacts associated with dust storms: a focus on the Middle East. Atmos. Environ..

[3] Sarver T, Al-Qaraghuli A, Kazmerski LL (2013). A comprehensive review of the impact of dust on the use of solar energy: history, investigations, results, literature, and mitigation approaches. Renew. Sustain. Energy Rev..

[4] Yilbas BS (2018). Environmental dust removal from inclined hydrophobic glass surface: avalanche influence on dynamics of dust particles. RSC Adv..

[5] Dubey P, Kaurav N (2019). Stoichiometric and Nonstoichiometric Compounds.

[6] Hugenholtz CH, Wolfe SA (2010). Rates and environmental controls of aeolian dust accumulation, Athabasca River Valley, Canadian Rocky Mountains. Geomorphology.

[7] Azarov AV, Zhukova NS, Kalyuzhina EA (2016). Environmental and working area dust emission from the gypsum warehouse. Procedia Eng..

[8] Yilbas BS (2015). Influence of dust and mud on the optical, chemical and mechanical properties of a pv protective glass. Sci. Rep..

[9] Lyu Y (2017). Characterization of dustfall in rural and urban sites during three dust storms in northern China, 2010. Aeolian Res..

[10] Hassan G, Yilbas BS, Said SAM, Al-Aqeeli N, Matin A (2016). Chemo-mechanical characteristics of mud formed from environmental dust particles in humid ambient air. Sci. Rep..

[11] Said SAM, Hassan G, Walwil HM, Al-Aqeeli N (2018). The effect of environmental factors and dust accumulation on photovoltaic modules and dust-accumulation mitigation strategies. Renew. Sustain. Energy Rev..

[12] Santhakumari M, Sagar N (2019). A review of the environmental factors degrading the performance of silicon wafer-based photovoltaic modules: failure detection methods and essential mitigation techniques. Renew. Sustain. Energy Rev..

[13] Ghosh S, Yadav VK, Mukherjee V (2019). Impact of environmental factors on photovoltaic performance and their mitigation strategies—a holistic review. Renew. Energy Focus.

[14] Ilse KK, Figgis BW, Naumann V, Hagendorf C, Bagdahn J (2018). Fundamentals of soiling processes on photovoltaic modules. Renew. Sustain. Energy Rev..

[15] Javed W, Wubulikasimu Y, Figgis B, Guo B (2017). Characterization of dust accumulated on photovoltaic panels in Doha, Qatar. Sol. Energy.

[16] Figgis B, Ennaoui A, Ahzi S, Rémond Y (2017). Review of PV soiling particle mechanics in desert environments. Renew. Sustain. Energy Rev..

[17] Ilse K (2019). Advanced performance testing of anti-soiling coatings—part I: sequential laboratory test methodology covering the physics of natural soiling processes. Sol. Energy Mater. Sol. Cells.

[18] Polizos G (2018). Anti-soiling and highly transparent coatings with multi-scale features. Sol. Energy Mater. Sol. Cells.

[19] Yilbas BS, Al-Sharafi A, Ali H (2019). Self-cleaning of Surfaces and Water Droplet Mobility.

[20] Sakarapunthip N (2017). Effects of dust accumulation and module cleaning on performance ratio of solar rooftop system and solar power plants. Jpn. J. Appl. Phys..

[21] Watanabe S (2018). MDC1 methylation mediated by lysine methyltransferases EHMT1 and EHMT2 regulates active ATM accumulation flanking DNA damage sites. Sci. Rep..

[22] Yong WYD, Zhang Z, Cristobal G, Chin WS (2014). One-pot synthesis of surface functionalized spherical silica particles. Colloids Surf. A Physicochem. Eng. Asp..

[23] Van Oss CJ, Good RJ, Chaudhury MK (1987). Mechanism of DNA (southern) and protein (western) blotting on cellulose nitrate and other membranes. J. Chromatogr. A.

[24] Heib F, Schmitt M (2016). Statistical contact angle analyses with the high-precision drop shape analysis (HPDSA) approach: basic principles and applications. Coatings.

[25] Cai J, Hao W, Zhang C, Yu J, Wang T (2017). On the forming mechanism of the cleaning airflow of pulse-jet fabric filters. J. Air Waste Manag. Assoc..

[26] Wilson H, VanSnick S (2017). The effectiveness of dust mitigation and cleaning strategies at The National Archives, UK. J. Cult. Herit..

[27] Swanson J-G, Langefeld O (2015). Fundamental research in water spray systems for dust control. Min. Technol..

[28] Yilbas BS (2016). Solvent-induced crystallization of a polycarbonate surface and texture copying by polydimethylsiloxane for improved surface hydrophobicity. J. Appl. Polym. Sci..

[29] Yilbas BS, Yousaf MR, Ali H, Al-Aqeeli N (2016). Replication of laser-textured alumina surfaces by polydimethylsiloxane: improvement of surface hydrophobicity. J. Appl. Polym. Sci..

[30] Ishida H, Koenig JL (1978). Fourier transform infrared spectroscopic study of the silane coupling agent/porous silica interface. J. Colloid Interface Sci..

[31] Nakabo S (2002). Regulation of fluoride ion release from Na2SiF6 contained in resin based on hydrophobic siloxane layer coating. J. Oral Rehabil..

[32] Nakamoto K (2006). Infrared and Raman Spectra of Inorganic and Coordination Compounds. Handbook of Vibrational Spectroscopy.

[33] Zhou L (2007). Transparent glass ceramic containing Er3+:CaF2 nano-crystals prepared by sol–gel method. Mater. Lett..

[34] Khunur MM, Risdianto A, Mutrofin S, Prananto YP (2012). Synthesis of fluorite (CaF2) crystal from gypsum waste of phosphoric acid factory in silica gel. Bull. Chem. React. Eng. Catal..

[35] Washburn EW (1921). The dynamics of capillary flow. Phys. Rev..

[36] Galet L, Patry S, Dodds J (2010). Determination of the wettability of powders by the Washburn capillary rise method with bed preparation by a centrifugal packing technique. J. Colloid Interface Sci..

[37] van Oss CJ, Good RJ, Busscher RJ (1990). Estimation of the polar surface tension parameters of glycerol and formamide, for use in contact angle measurements on polar solids. J. Dispers. Sci. Technol..

[38] Zhang X, Wang X, Miller JD (2015). Wetting of selected fluorite surfaces by water. Surf. Innov..

[39] Kim D, Pugno NM, Ryu S (2016). Wetting theory for small droplets on textured solid surfaces. Sci. Rep..

[40] Anand S, Rykaczewski K, Subramanyam SB, Beysens D, Varanasi KK (2015). How droplets nucleate and grow on liquids and liquid impregnated surfaces. Soft Matter.

[41] Bergeron V, Langevin D (1996). Monolayer spreading of polydimethylsiloxane oil on surfactant solutions. Phys. Rev. Lett..

[42] Carlson A, Kim P, Amberg G, Stone HA (2013). Short and long time drop dynamics on lubricated substrates. EPL Europhys. Lett..

[43] Yilbas BS (2019). environmental dust particles repelling from A Hydrophobic surface under electrostatic Influence. Sci. Rep..

[44] Butt H-J, Cappella B, Kappl M (2005). Force measurements with the atomic force microscope: technique, interpretation and applications. Surf. Sci. Rep..

[45] Jańczuk B, Wójcik W, Zdziennicka A (1993). Determination of the components of the surface tension of some liquids from interfacial liquid-liquid tension measurements. J. Colloid Interface Sci..

